# Research on the Influence of the AW 5754 Aluminum Alloy State Condition and Sheet Arrangements with AW 6082 Aluminum Alloy on the Forming Process and Strength of the ClinchRivet Joints

**DOI:** 10.3390/ma14112980

**Published:** 2021-05-31

**Authors:** Jacek Mucha, Ľuboš Kaščák, Waldemar Witkowski

**Affiliations:** 1 Faculty of Mechanical Engineering and Aeronautics, Rzeszów University of Technology, 35-959 Rzeszów, Poland; wwitkowski@prz.edu.pl; 2 Department of Technology, Materials and Computer Supported Production, Technical University of Košice, 040 01 Košice, Slovakia; lubos.kascak@tuke.sk

**Keywords:** clinching, clinch riveting, AW5754 and AW6082 aluminum alloys, joint tensile shear strength, AW5754 aluminum alloy state

## Abstract

Clinching joints with an additional deformable rivet are modifications of the clinching joints. The clinch riveting (CR) joint is formed indirectly by a deformable rivet. The research included an analysis of CR joints’ forming process for aluminum alloy sheets made of AW 6082 in T6 state condition and AW 5754 in three different state conditions: H11, H22 and H24. As a result of forming the joint for various sheet arrangements, the highest value of blocking the upper sheet in the lower sheet (*t_u_*) was obtained for the arrangements with two 5754-H24 aluminum alloy sheets. For such a large interlock parameter *t_u_*, the greatest thinning of lower sheet (*t_n_*) was obtained, which influenced the maximum tensile shear force and the joint failure mechanism. Based on the load-displacement diagrams obtained from the static shear test of lap joints, the total energy of failure and energy to achieve the maximum load capacity were calculated. The highest energy absorption to achieve the maximum load capacity, in the case of the same sheet materials, was obtained for the 5754-H11 aluminum alloy sheets. On the other hand, among the tested combinations, the highest value of energy absorption (for the joint maximum load capacity) was obtained for the sheet arrangement: top sheet AW 6082-T6 and the bottom AW 5754-H24. The highest value of the total energy up to fracture was obtained when the material of the top sheet was AW 6082-T6, and the bottom AW 5754-H22. For each sheet arrangement, a similar analysis of the joint strength parameters, interlock parameters and forming force were made.

## 1. Introduction

In the automotive industry, aluminum sheet metal is commonly used to create a lightweight car body assembly. All individual parts of the body structures need to be joined. The most commonly used joining methods for aluminum parts are welding, resistance welding, brazing, soldering and friction stir welding. Other joining methods that can be used in the assembly process of aluminum elements are adhesive bonding, fastening, riveting, clinching, clinch riveting, self-piercing riveting or tabs bending [1,2]. In welding processes, the thermal after-effects and distortion are obtained [3,4]. Joints produced by adhesive bonding overcome the welding difficulties, even in joining sheets made from dissimilar materials, but their use is constrained by temperature, ageing due to weathering, long curing times and precision of surface preparation. The mechanical fastening and joining by pressing (clinching, clinch riveting self-piercing riveting, tabs bending) are free from thermal after-effects. These methods can be also used to join metallic and non-metallic sheets. Fastening, with the use of bolts or rivets, usually requires making a hole for having access from both sides of the connected elements. Joining by the pressing method usually does not require an additional hole. The use of pressed joints is limited by the maximum joining force, the sheets’ material elastic spring back, the protrusion of the joint and sheet thickness [1,2]. Joining by pressing methods are still being developed and improved, so a brief account of new varieties of pressing joints (clinching, self-piercing riveting, clinch riveting) will be presented in this paper [5].

Clinching joining process, with the use of punch and die (rigid or with movable segments), consists in producing an interlock for two or more metal sheets. In this process, the geometric interlock, also called the S-shape, is obtained. The main geometric parameters of the clinched joint are thickness of the embossment (parameter *X*), width of the interlock in the upper sheet (*t_n_*), width of the interlock in the lower sheet (*t_d_*) and interlock angle (*θ*). The designed groove in the die controls sheet material flow, and it is critical to the formation of the interlock [1,2]. Witkowski [6] presented the strength of clinching joints made by rigid tool for two sheet material: steel DX51D + Z/275 and the aluminum alloy EN AW-5754 in O/H111 state. The shear, peel and tension performances and force-displacement curves were compared. Lei et al. [7] studied the clinch-bonded joints’ strength in tensile shear and peeling tests for combination of copper (H62), aluminum alloy (AW 5052) and steel (Q215) sheet arrangements. The adhesive layer improved the hybrid joints’ shear strength. The joint peeling strength was determined by clinching joints. For the 5754 aluminum alloy in O/H111 state, Mucha et al. [8] determined the strength during multi-axis load condition (from shear test to tension test with the angle changing 15° between load force and joint axis). Chan-Joo et al. [9] proposed a method for designing rigid tools that join Al6063 alloy sheets by the clinching method. The joints’ strength was calculated numerically by using geometric parameters of the clinched joints obtained in FEM analysis. Experimental top-hat impact test was carried out for mechanical clinched joint. The obtained results were similar to the SPR joints’ strength.

Lambiase [10] presented an analysis of the tools’ geometry modifications and aluminum alloy sheets’ pre-heating influence on the material flow in the clinching process. The AA6082-T6 sheets were joined by clinching using die with movable segments. He concluded that the development of cracks at the interlock can be prevented by pre-heating the joined sheets. Moreover, pre-heating allowed to produce joints without cracks for sheet materials with low ductility. The changes in die geometry (different die anvil depths) allowed to prevent the formation of cracks at room temperature. Pre-heating caused the decreasing of the local hardness of the material (increase in grains). The development of a hot stamping clinching tool was presented by Li-Wei et al. [11]. A traditional heating furnace was replaced by a resistance heating system containing heating, forming and colling systems.

Another method for increasing clinching joints’ strength is reforming joints. Chao et al. [12] investigated reforming force, failure process, interlock parameters, joint strength and joint energy absorption of the reformed clinching joints for AL6061-T4 sheets. Three different reforming methods were used in their study: special-rivet use, no-auxiliary use and bumped-die use. For all reforming methods, cross-tension strength, tension-shearing strength and energy absorption increased.

Die-less clinching process is a relatively new process variety of clinching joining technology. It was developed in order to make clinching joints with protrusion on one side. The flat anvil is used instead of the die with grove. The material flow in a designed groove in the blank holder. Sabra Atia et al. [13] presented a finite element modeling analysis of the influence of material and tool stiffness conditions on die-less clinch joining method characteristics. The main goal was to clarify and optimize die-less clinching numerical simulations for improved and efficient joints’ interlock predictions. The analyzed numerical parameters were mesh element size and its aspect ratio, adaptive remeshing criterion, rigid tool stiffness, material parameters (hardening laws) and friction between all elements. The size of the finite elements was a critical parameter for the prediction of geometrical parameters of interlock shape. Adaptive re-meshing technique had no effect on the interlock shape. The material model, due to very large plastic strains, was an important issue in the simulation. The spring-back effect was depended on the friction coefficient. The material flow behavior and interlock parameters during the die-less clinching process were investigated by metallographic observations by Sabra Atia et al. [14]. Blank holders of different shapes and dimensions were used to control the material flow of 7075 aluminum alloy sheets. The sheet material was in different temper conditions: O, W and T6. The temper states of sheets had a significant influence on the clinch-ability and interlock parameters. Shear strength of the die-less clinched joints depended on the interlock parameters: width of the interlock in the upper and lower sheets. Peeling joint strength depended on the width of the interlock in the upper sheet. Gerstmann et al. [15] studied the flat clinch-bonding process (flat-clinching and adhesive bonding) for 1050A aluminum alloy sheets. Results from experimental tests were implemented to the simulation model. The adhesive pockets’ influence on the interlock parameters was that they prevented interlock formation. 

Lin et al. [16] proposed the use of friction stir clinching (FSC) for joining alclad AA2024-T3 sheets, which are difficult for the welding process. They correlated fatigue data of FSC joints with effective stress intensity factor ranges. For tensile-shear and cross-tension samples of the joint strength, critical fatigue cracks and failure modes were obtained and identified. Zhou et al. [17] used modified friction stir clinching (MFSC) for 5754-O and 2024-T3 aluminum alloy sheets. The sheet arrangements strongly influenced the joints’ strength.

Zhang et al. [18] used a resistance spot clinching (RSC) method—combination of mechanical clinching and resistance spot welding processes—for joining 5052 aluminum alloys. The fusion zone of a resistance spot clinching joint consisted of a larger equiaxed zone compared with a conventional resistance spot welding joint. The high stiffness of the RSC joint led to a higher tensile strength. The resistance spot clinching compared with the traditional resistance spot welding process is more energy efficient. For joining 5754 aluminum alloy with drawing-quality special-killed (DQSK) steel sheet, a high energy absorption (three times more than that of spot-welded joint) was obtained [19].

Babalo et al. [20] presented a new joining process: electro-hydraulic clinching (EHC). The shock wave in the fluid, caused by electrical energy discharge, forms the clinching joint. The obtained joint efficiency was about two times higher than that of the conventional clinching process. Wang et al. [21] proposed a novel method of clinching joining —incremental laser shock clinching. This method can be used for forming joints as a rectangular (the joint length is larger than laser spot diameter). Aluminum alloy parts can be also joined by pressing with the use of additional rivet: self-piercing riveting [22] and clinch riveting. Mucha et al. [23] presented the pressed joint technology using forming process without (clinching) or with additional fastener (clinch riveting). An increase in the load-carrying ability of clinching joints by applying special rivets and dies was obtained. 

Huang et al. [24] studied the fatigue behavior of self-piercing riveted joints of aluminum alloy 6111-T4. They determined the dominant failure mode (the corner crack). From experimental and numerical analysis, they propose a new approach in determining crack growth.

Wood et al. [25] present the performance of self-piercing riveted joints in 5754 aluminum alloy sheets at low rate and at typical automotive crash speeds. The effect of test speed on the joint peel performance (strength, displacement and failure mode) between the heat-treated specimens and non-heat-treated specimens was negligible. The low-rate test results for the three joint types with and without heat treatment were the same: the energy dissipated was improved for the joint specimens with heat treatment, maximum load force was highest in shear and the lowest in peel.

Li et al. [26] studied the mechanisms of crack initiation and growth during fatigue strength test for self-piercing rivet joints. The influence of fatigue on the stiffness and static strengths of SPR joints was obtained for 5754-PT2 aluminum alloy sheets. The influence of the friction of 5754 aluminum alloy sheets with different surface textures was presented by Li et al. [27] The different surface textures did not have a significant influence on the joining process (forming forces, interlock dimensions).

Cai et al. [28] presents the research of the assembly dimensional prediction area, using experimental data and finite element analysis results, for SPR and RSW joint distortion. The joining material was 5754 aluminum alloy. The joint local distortion due to self-piercing riveting was bigger than that of the resistance spot welding joints. 

Ma et al. [29] investigated the tensile shear strength and fracture mechanism of the friction self-piercing riveting technology (F-SPR: combination of self-piercing riveting and friction stir spot welding processes) for 7075-T6 aluminum alloy sheets. The observed fracture mechanisms were rivet shear and pull-out. This joining method can be used for low-ductility materials. Two fracture modes were observed: rivet shear fracture and rivet pull-out fracture. In [30], Ma et al. studied F-SPR joint strength for combination of sheet materials: aluminum alloy, low-ductility magnesium alloy and high-strength steel. Paidar et al. [31] used the modified friction stir clinching (MFSC) process for joining 2024-T3 and 6061-T6 aluminum alloy sheets. The tensile joint strength and changes in microstructures of joined materials were depended on the sheet arrangement. Ying et al. [32] proposed joining aluminum with high strength and low ductility (such as AA7075-T6) by the SPR joining technology with pre-heating of the sheets, increasing the value of temperature influence on the interlock geometry and joints’ strength. There are only few studies with the results of research on the formation process and strength of clinched joints made with an additional deformable full rivet (CR—clinch riveting). The authors presented a new joining method, other than clinching or SPR technology, with the use of a solid deformable rivet for joining 6082 aluminum alloy sheets (in T6 state condition) with 5754 aluminum alloy sheets (in three different state conditions: H11, H22, H24). The influence of material arrangements for sheets made of 6082 and aluminum alloy on the joints’ strength was obtained. An analysis of the influence of the aluminum alloy 5754 states on the forming force and joint strength were performed. The measurement results on the cross-section of the joint with the full deformable rivet were also presented.

## 2. Materials and Methods

### 2.1. Experimental Materials

Two types of aluminum alloys: EN AW 5754 (AlMg3, WNR: 3.3535) and EN AW 6082 (AlSi1MgMn, WNR: 3.2315) were used in the forming process of clinching joints with an additional full rivet. The materials of joined sheets are characterized by mechanical properties and state conditions (Table 1). The sheet thicknesses were 0.8 mm (AW 5754) and 0.9 mm (AW 6082). Hence, on force-displacement diagrams, the *s_i_*/*s_max_* displacement index was used. 

**Figure 1:**
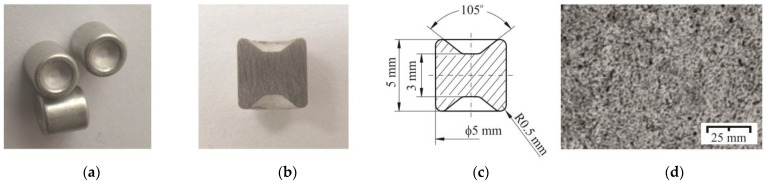
Additional rivets used in clinching forming process: (**a**) real view, (**b**) axial section, (**c**) basic dimensions, (**d**) rivet material microstructure.

Tox Pressotechnik rivets with A5x5-2Al catalog number were used in clinching joining process as an additional deformable element (Figure 1a–c). The average hardness of the steel rivets for the five measurements was 345 HB. The basic geometry is shown in Figure 1c. The microstructure of the rivet material was presented in Figure 1d.

### 2.2. Experimental Procedure

The sheet metal specimens were cut from the aluminum alloy sheets for each material state condition. For each sheet arrangement, five samples were prepared—to determine the average joint forming force and the average tensile shear force. The list of sheet material arrangements was presented in Table 2. 

**Figure 2:**
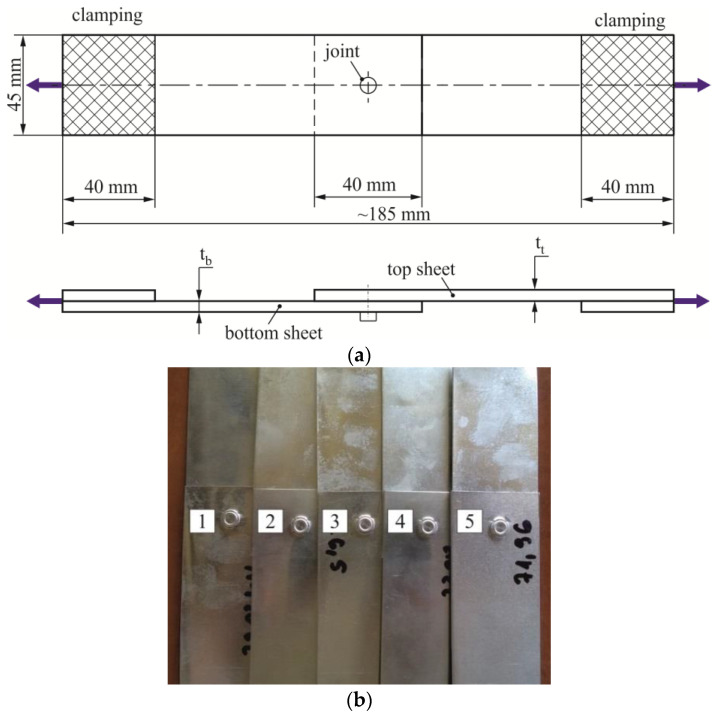
Single-lap tensile shear test specimens: (**a**) dimensions, (**b**) five samples for one sheet arrangement.

In previous publications, the size of the overlap is assumed in various ways. For example, Xu [33] used overlapping of 50 mm for SPR joints for 5754 aluminum alloys sheets. In another work [34] by He et al., the size of the overlapping was specified as 20 mm for the 5052 aluminum alloy sheets. In another publication [35] an overlap of 40 mm was assumed for sheets made of 5052 aluminum alloy. For hybrid resistance spot clinching and 5052 aluminum alloy sheets, the overlap was measured 25 mm [18]. For our tests, the sample geometry, including the size of the overlap, was determined on the basis of the guidelines contained in ISO 12996: 2013 standard [36]. The dimensions of single-lap joints for tensile shear tests were presented in Figure 2a, and five samples for one arrangement were shown in Figure 2b. 

**Figure 3:**
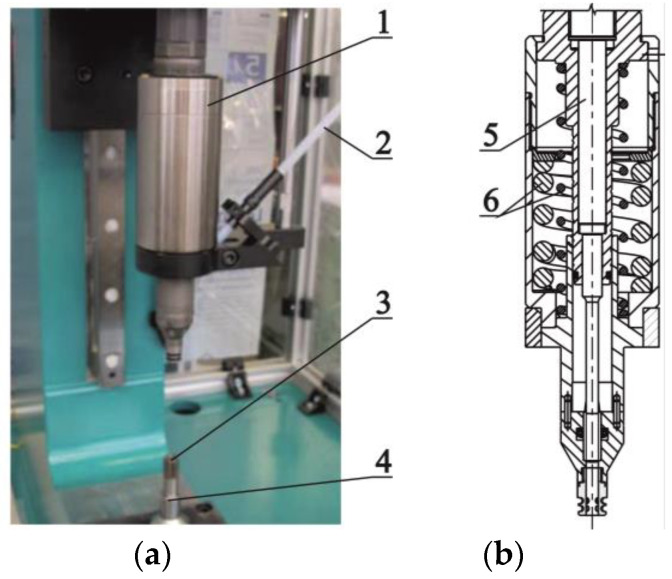
Forming tools used in the CR joining process: (**a**) punching system with rivet feeding system, (**b**) spring system integrated with punching system (1-frame, 2-rivet feeding tube, 3-die with movable segments, 4-die holder, 5-punch, 6-spring system).

**Figure 4:**
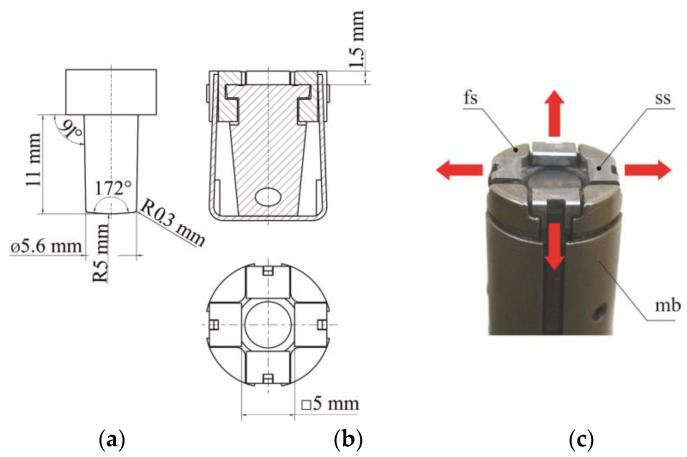
Clinch-riveting tools: (**a**) punch, (**b**) extensible die, (**c**) fixed and movable segments (fs—fixed segment, ss—sliding segment, mb—die base).

The joint forming tools (Figure 3a) was mounted on the Tox Pressotechnik frame construction press type CMB with maximum force load 100 kN and electric drive EMPK. The punch system (no.1 on Figure 3) was mounted to the vertically moved drive, and the TOX SKB 14.180.246182 die (no.3 on Figure 3) was mounted with the holder (no.4 on Figure 3) to the bottom part of the c-frame press. In the punching system, a rivet feeding system and a spring system are integrated with the punch movement system (Figure 3b). In clinch-riveting joining process, a die with movable segments were used. The main dimensions, the die geometry and the direction of segments’ movements were presented in Figure 4. For all samples, the pressing movement was realized until the rivet was leveled with the surface of the upper sheet. The punch positioning accuracy was 0.01 mm, and the maximum error of the measured forming force was 0.5% of the force.

**Figure 5:**
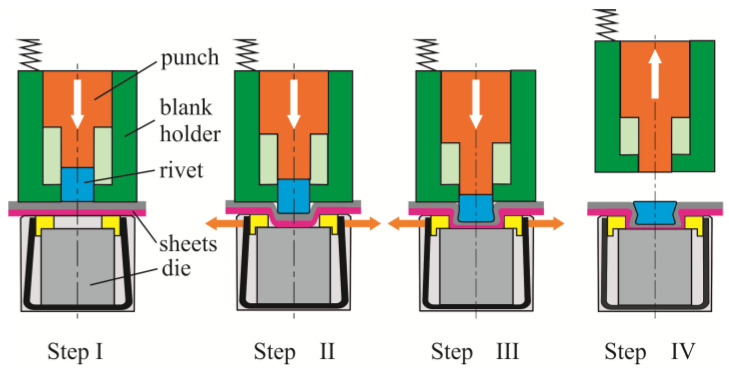
Steps of the ClinchRivet joining process.

The forming process for each forming step was presented in Figure 5. During forming the ClinchRivet joint, the embossment and the interlock is indirectly formed by an additional deformable rivet. After the lower sheet touches the die bottom, the rivet material flows radially (during its upsetting). The pressure of the blank holder and spring system causes the pressing on the movable and fixed segments of the die. After reaching the boundary friction force between the surface of the movable segments and the sheet surface, the movable segments move radially to the movement of the punch. The material of the joined sheets flows, and the rivet is blocked in their material at the same time. After reaching the position of the punch face with the upper surface of the sheet, the tool is retracted. In this way, the ClinchRivet joints are obtained.

**Figure 6:**
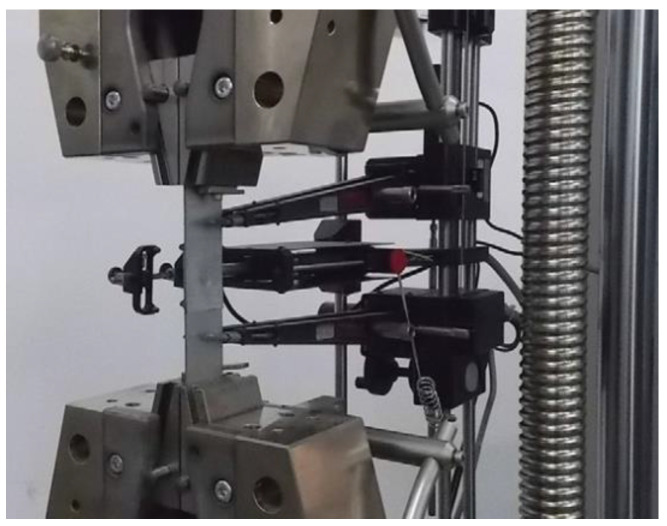
The extensometer’s measuring system used in tensile shear tests.

**Figure 7:**
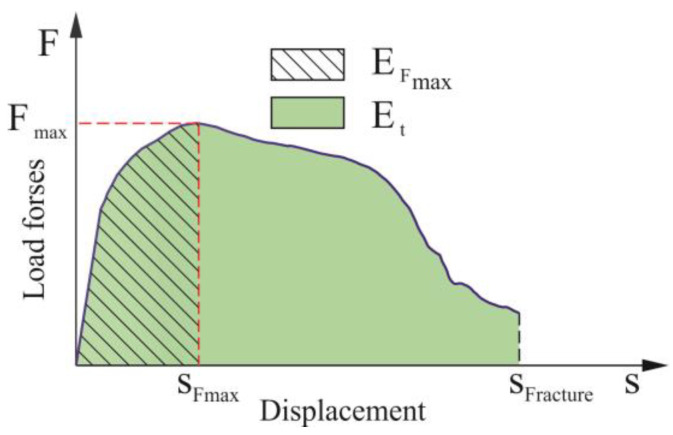
Load-elongation diagram for the tensile shear test and dissipated energy.

The tensile shear tests were performed for single-lap joint specimens in accordance with the guidelines from ISO 12996: 2013 [36]. The static shearing test was performed using Instron 3382 machine. The traverse speed of the testing machine was set up to 10 mm/min. The extensometer’s measuring system (Figure 6) was used to measure the displacement. The measuring arm’s space was 50 mm. According to the ISO 12996: 2013 standard, the selected parameters (Equations (1) and (2)) from the joint strength tests were determined (Figure 7).

(1) $$E_{t}=\int_{s = 0}^{s_{Fracture}}F\cdot ds$$

where:

*E*_t_ [J]—dissipated energy up to fracture,
*F* [N]—tensile shear force,
s [m]—displacement,
*s_Fracture_* [m]—total displacement up to fracture,

(2) $$E_{F_{\max}}=\int_{s=0}^{s_{F_{\max}}}F\cdot ds$$

where:

*E_F_*_max_ [J]—dissipated energy up to maximum tensile shear force,
*F* [N]—tensile shear force,
s [m]—displacement,
*s_F_*_max_ [m]—displacement at maximum tensile shear force.

## 3. Results and Discussion

### 3.1. ClinchRivet Joint Forming Process

**Figure 8:**
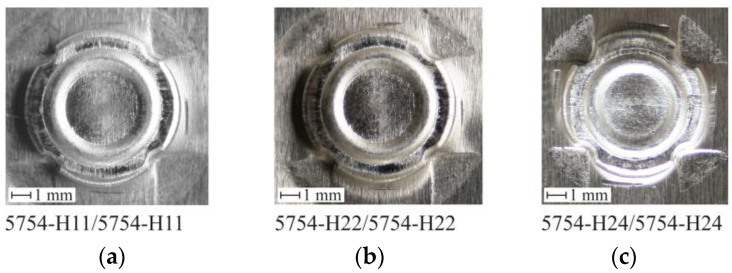
The embossments obtained during the joint forming process for identical materials of the top and bottom sheets. Sheet arrangement in accordance with Table 2: (**a**) no.1, (**b**) no.4, (**c**) no.7.

The CR joint forming tests were carried out for the materials in the arrangements presented in Table 1 (in three groups). In each case, no lubricant was applied to the forming tools. For joints made for AW 5754 materials and three different state conditions H11, H22, H24, no cracks were observed on the embossing side (Figure 8), and the cohesion of the sheet material was preserved. In the case of the AW 5754 material in H24 state condition, the highest material flow was obtained beyond the fixed die segments (Figure 8c).

**Figure 9:**
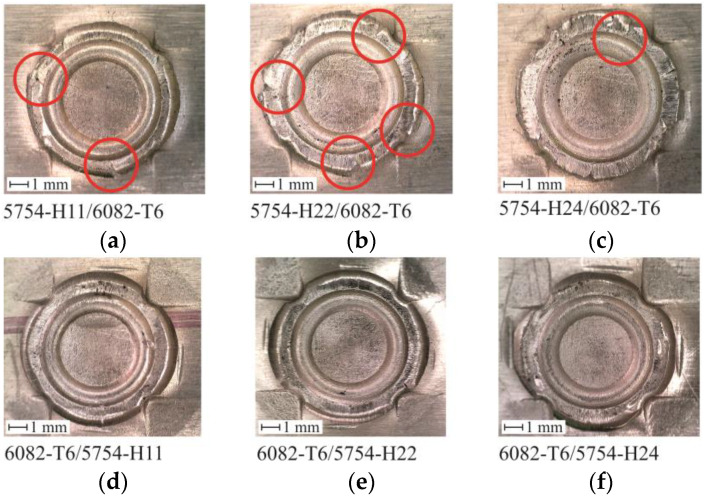
The embossments obtained during the joint forming process with AW 6082-T6, AW 5754-H11, AW 5754-H24 and AW 5754-H24 different arrangements. Sheet arrangement in accordance with Table 2: (**a**) no.2, (**b**) no.5, (**c**) no.8, (**d**) no.3, (**e**) no.6, (**f**) no.9.

In all other arrangements of sheet material, for which the lower sheet was made of the 6082-T6 aluminum alloy, slight surface cracks were observed in the areas of free plastic deformation of the material (the area of free material movement between the fixed die segments)—Figure 9a–c. In the case of the joints, when the upper plate was made of AW 5754-H11 and the lower plate was made of AW 6082-T6, cracks were observed only in two areas; these cracks did not occur along the entire depth of the material of the lower sheet. In the other two cases (Figure 9b,c), it was difficult to determine at what depth the crack had occurred. The use of the material AW 6082-T6 on the upper sheet, and the AW 5754 on the lower sheet with the three state conditions H11, H22, H24 did not cause any cracks on the embossment side (Figure 9d–f). AW 5754 aluminum alloy in three cases of the state condition maintained the continuity of the material in the areas of free flow of the material (between the fixed segments of the die). 

**Figure 10:**
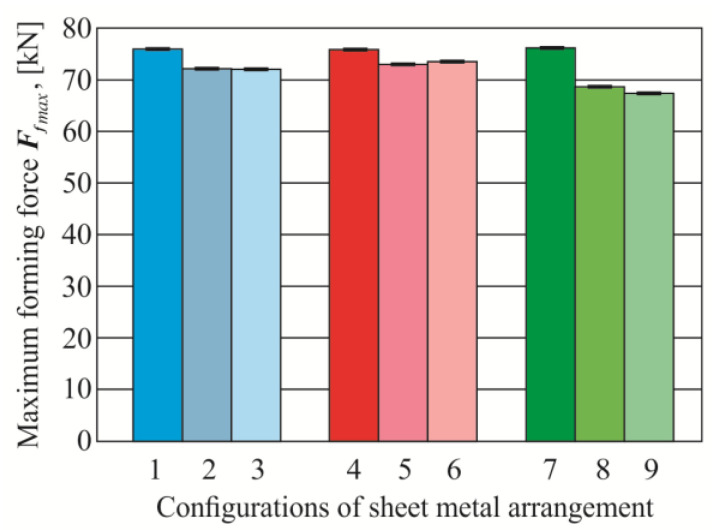
The average values of maximum joint forming force.

The biggest impact of changing the material arrangement of the upper and lower sheets on the forming force occurred in the case of a change in the identical arrangement of sheets made of 5754 aluminum alloy in the different state condition H11, H22, H24 in combination with the AW 6082-T6—Figure 10. In the case of joining sheets of 5754 aluminum alloy, in a different state condition, the maximum forces of CR joint forming were similar. In the third group of material arrangement of the sheets’ material AW 5754-H24 and AW 6082-T6, the biggest effect of the sheet arrangement on the maximum forming force of the CR joint was obtained. In the first group of the sheet arrangement, the change of the AW 6082 material from the lower to the upper did not change the forming force (bars 2 and 3 in Figure 10). The maximum forming force for joints with the no.9 arrangement of sheet material was 68 kN, while, for the reverse system (arrangement no.8), it slightly increased the force to 69 kN (increase of about 1.5%).

**Figure 11:**
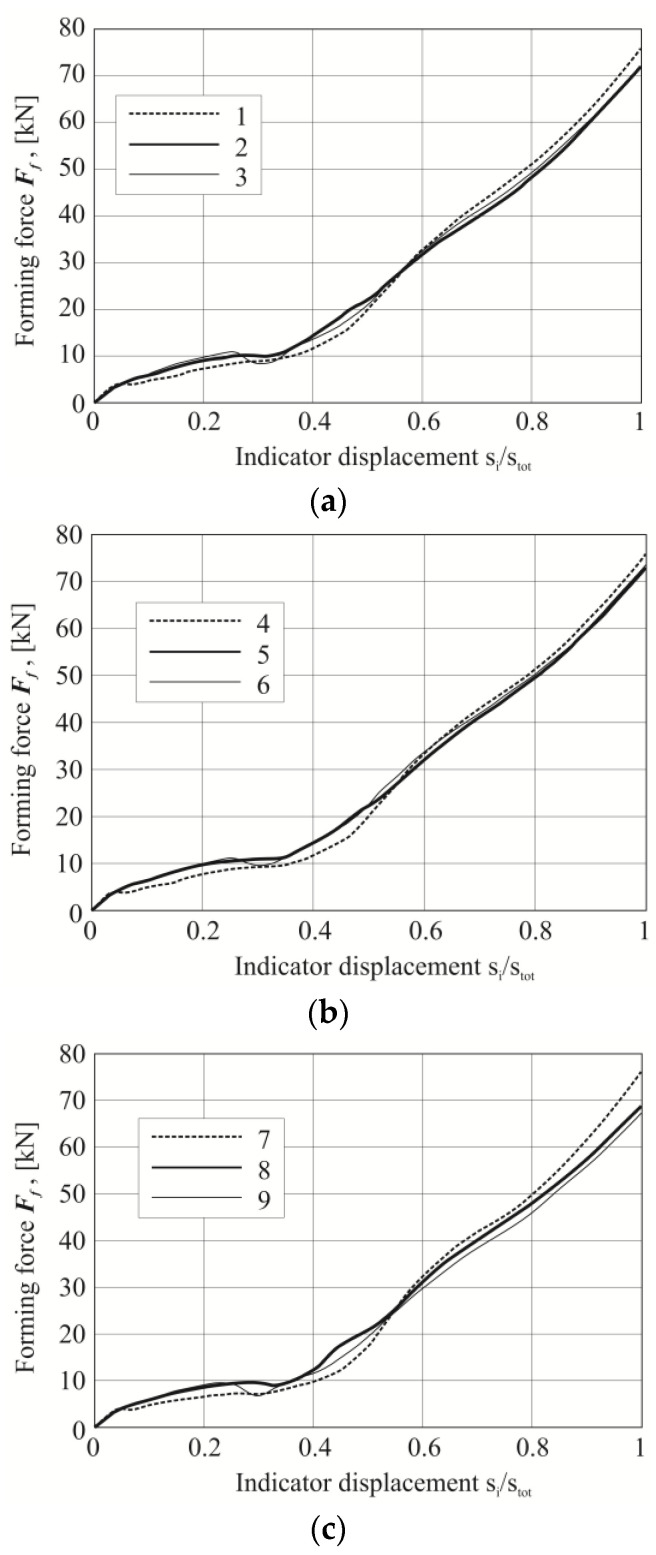
The forming force-displacement diagram for three group of the sheet arrangements (**a**) AW 5754-H11 and AW 6082, (**b**) AW 5754-H22 and AW 6082, (**c**) AW 5754-H24 and AW 6082.

**Figure 12:**
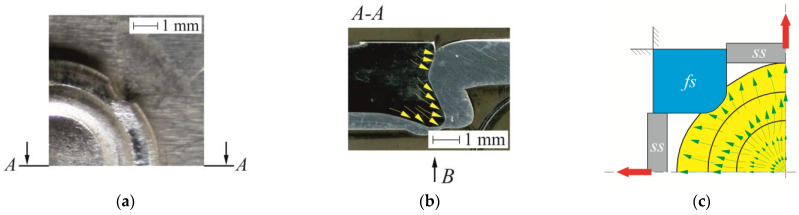
The characteristic of CR joints: (**a**) fragments of the embossment (from die side), (**b**) material flow directions of the rivet material in cross-section view, (**c**) material flow directions in relation to the fixed and movable segments of the die.

**Figure 13:**
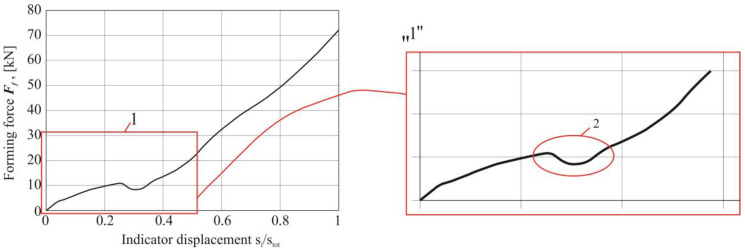
The forming force-displacement diagrams with the characteristic phase of the intensive movement of the rivet in the radial direction (sheet arrangements: AW 5754-H24 and AW 6082).

Additionally, a comparative analysis of the registered characteristics of the joints’ forming force was performed for the sheet material arrangements (Figure 11). Three curves were compiled on separate charts, respectively, for each group of material arrangement of the upper and lower plates (as in Table 2). Depending on the AW 5754 state and the change in the arrangement of the sheet material, different diagrams of the forming force were obtained. The initial linear part resulted from the compliance characteristics of the forming tools. Significant differences in the course of the curves were observed for the value of the displacement index (*s_i_*/*s_tot_*) from 0.25 to 0.55 (Figure 11). The rivet was pressed into the materials until the lower metal sheet touched the bottom of the die (Figure 12a–c). Subsequently, further pressure of the punch on the rivet caused its upsetting and the flow of material in the radial direction (Figure 12b). The initial plastic flow of the material (with the simultaneous slight rotation of the moving segments) caused a local decrease in the forming force (detail “2” in Figure 13). Further pressing of the rivet caused the deformed sheet material to be pushed outside the die (between fixed segments), until the displacement index of about 0.55 was reached (Figure 11). Further pressing caused the rivet material and the sheets to flow between the fixed segments (Figure 12c), but with ever-greater force (the movable segments rotated and allowed the material to flow). At this stage, until the maximum displacement of the punch was achieved, the rivet and the sheets to be joined were interlocked. A similar material flow mechanism during the joint formation for self-piercing riveting technology has been described by Haque et al. [37]. It should be noted that when using CR technology, a die with movable segments is used. In the SPR technology, there is no such intense radial movement of the sheet material as in the case of forming of the CR joint. Due to the deformation of the rivet (rivet upsetting), the material of the joined sheets is forced to flow. As a result, an interlock is formed. In the SPR technology, the interlock is created as a result of piercing the upper layer or layers and a partial piercing of the bottom layer. The CR rivet has two identical groves (Figure 1b). When the rivet is pressed in, the material of the rounded edge of the rivet (Figure 1c—outer part of the grove) flows in a radial direction.

The CR technology requires the use of die with a complex construction. Intensive research is being done to simplify the construction of the die for the clinching process. It is now possible to form a joint using a flat die. Sabra Atia et al. [13,14] provided the possibility of clinching joining for sheet material of 7075 aluminum alloy sheets. They showed how the forming force influences the increase in the maximum strength of clinching joints formed with a flat die. 

### 3.2. ClinchRivet Joint Strength

The flattest tensile shear force-displacement diagram was obtained for the specimen with no.9 sheet arrangements according to Table 2. The highest value of the load (*F_s_*) was obtained for the highest value of the displacement (*s_Fmax_* = 3.5 mm). For this sheet arrangement, the proportionality range changed quite intensively into a non-proportional curve. The highest repeatability of proportionality range, for five tensile shearing tests, was obtained for no.8 and no.9 sheet arrangements. In the paragraph, the dissipated energy up to fracture, up to maximum tensile shear force and up to 0.3 of maximum tensile shear force were determined in accordance with ISO 12996 [36].

Among all sheet arrangements, the highest joint stiffness in the linear range was obtained for the no.2 arrangement. However, it was quickly changed as the load increased.

**Figure 14:**
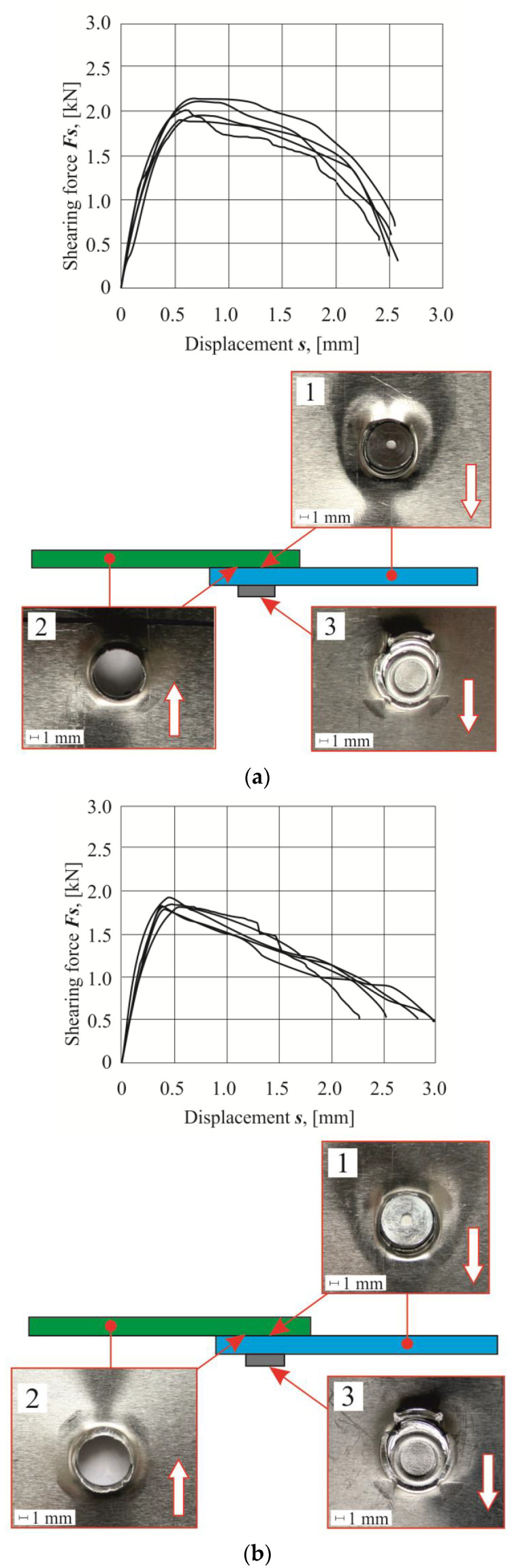

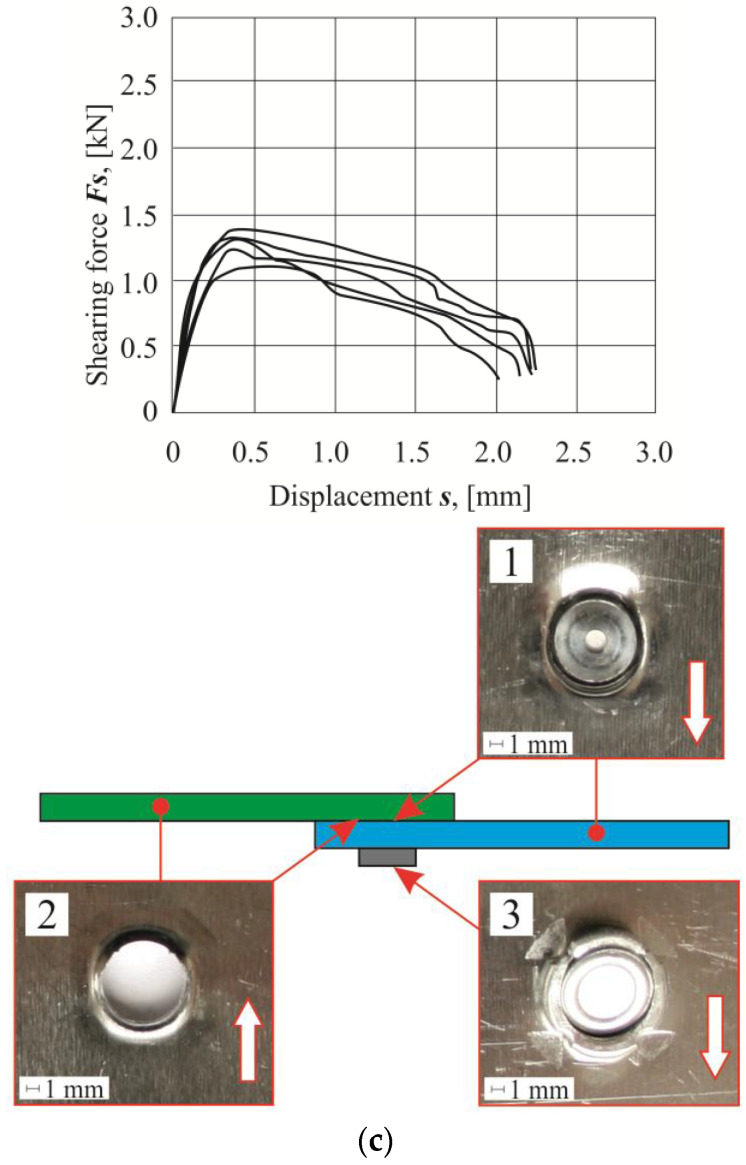
The tensile shear force-displacement diagrams (left side) and joint fragments after tensile shear test: (**a**) arrangement no.1 (AW 5754-H11 with AW 5754-H11), (**b**) arrangement no.4 (AW 5754-H22 with AW 5754-H22), (**c**) arrangement no.7 (AW 5754-H24 with AW 5754-H24).

**Figure 15:**
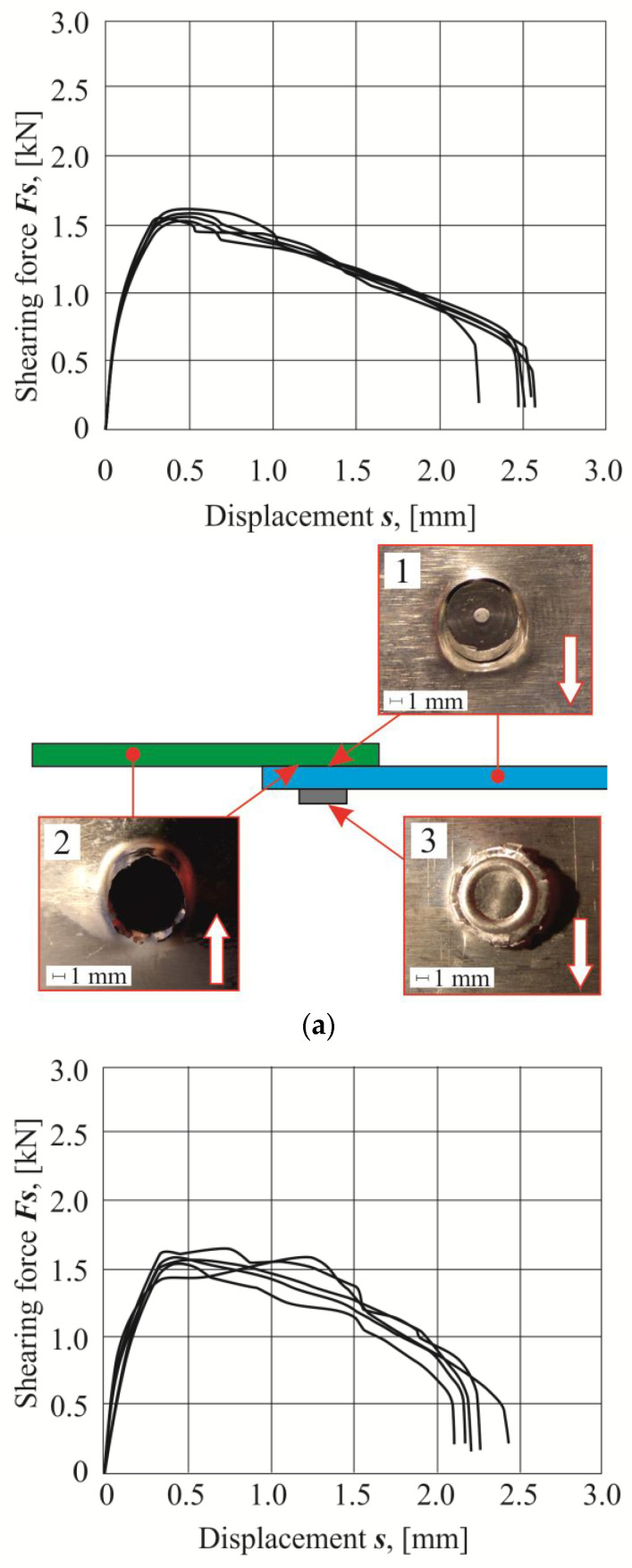

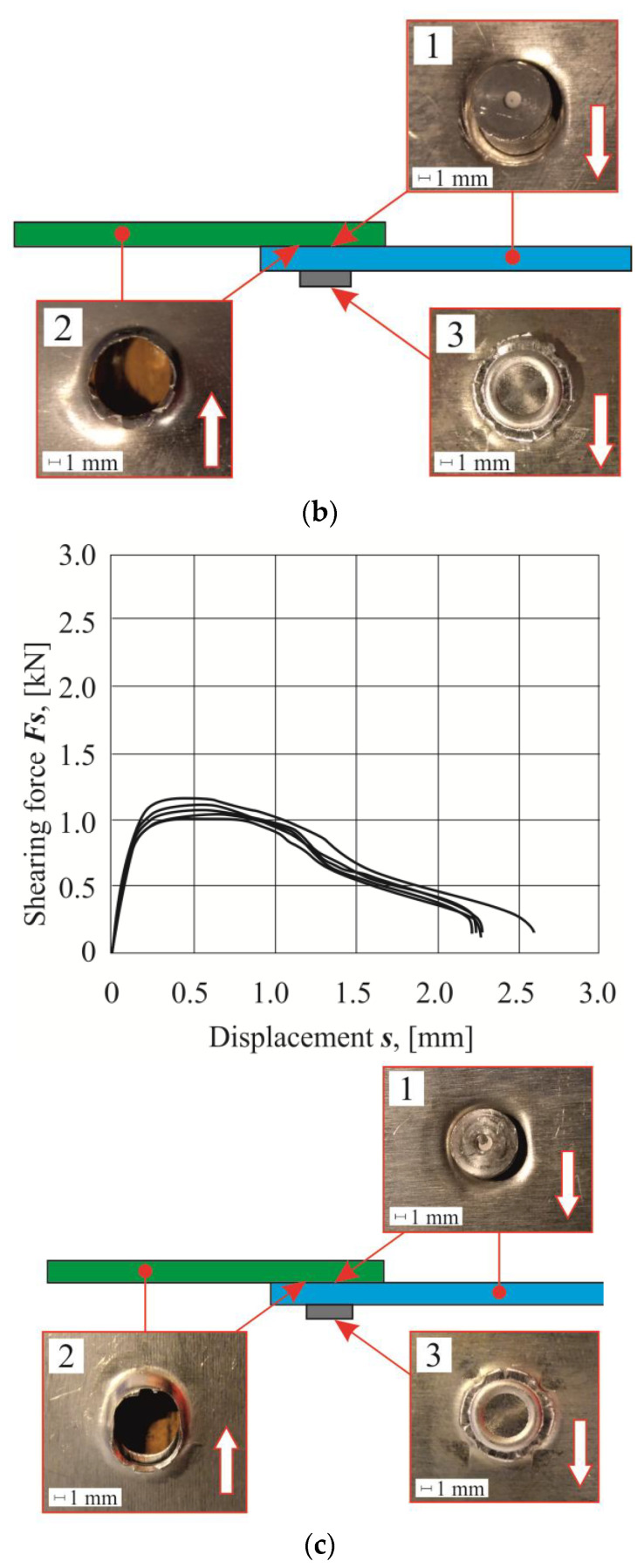
The tensile shear force-displacement diagrams (left side) and joint fragments after tensile shear test: (**a**) arrangement no.2 (AW 5754-H11 with AW 6082), (**b**) arrangement no.5 (AW 5754-H22 with AW 6082), (**c**) arrangement no.8 (AW 5754-H24 with AW 6082).

**Figure 16:**
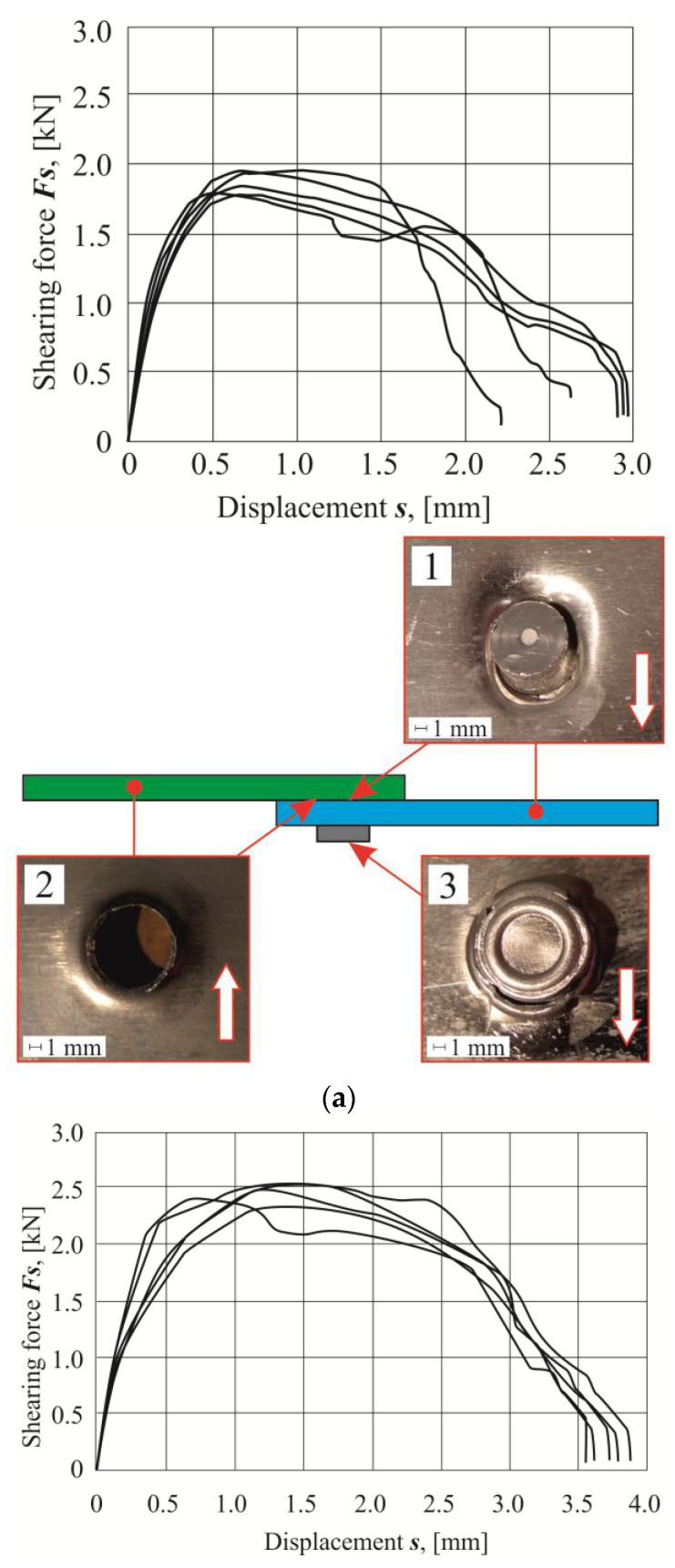

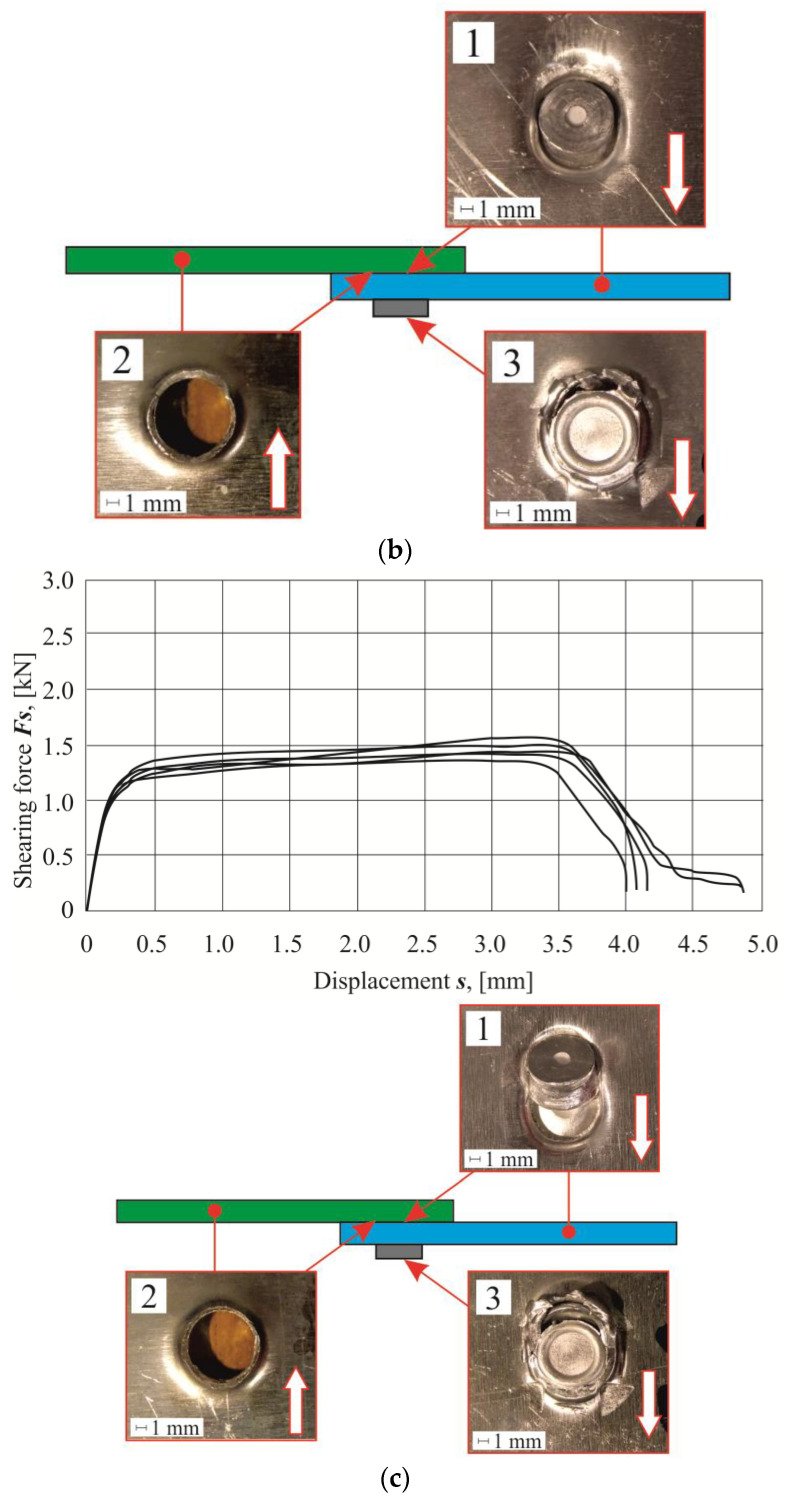
The tensile shear force-displacement diagrams (left side) and joint fragments after tensile shear test: (**a**) arrangement no.3 (AW 6082 with AW 5754-H11), (**b**) arrangement no.6 (AW 6082 with AW 5754-H22), (**c**) arrangement no.9 (AW 6082 with AW 5754-H24).

For each arrangement of the sheet materials, a list of the obtained shear diagrams of the lap joints from five tests was presented (Figure 14, Figure 15 and Figure 16). Based on the load force-displacement diagrams and on the observation of the failure mechanism, it can be concluded that certain phases of joint deformation occur. As in the case of the SPR joints [38], in the CR joints, the large plastic deformations were obtained. In the case of joints with the sheet material arrangement no.1, 4, 7 and 8, ductile failure occurred without rivet rotation. On the other hand, for the joints with the sheet material arrangement no.2, 3, 5, 6 and 9, there was a ductile failure after partial rotation of the rivets. The largest displacement to achieve the maximum shear force was obtained for the arrangement no.9. In this case, during the shear of the CR joint, the rivet was gradually pulled out until the critical value of the ductile deformation of the upper sheet material was achieved.

One of the methods of ensuring that the rivet will not be pulled out is the use of modified CR technology. Some modification of the CR technology by rivet welding was made by Zhang et al. [19]. The use of an additional rivet in the welded joints increased the strength and the dissipated energy of the joints.

It should be mentioned that the AW 6082 aluminum alloy in the T6 state condition had the highest yield strength and initial hardness among the materials used (Table 1). Depending on the material state condition and the arrangement of the sheets, during the shear test, the joint area was deformed to a greater or lesser extent (photos on the right in Figure 14, Figure 15 and Figure 16). In all cases, the interlock strength of the upper sheet was less than the material strength around the rivet. Hence, as a result of the tensile shearing test, the destruction was caused by the loss of cohesion of the material on the cross-section of the interlock formed in the upper sheet (photo 2 in Figure 14, Figure 15 and Figure 16). Analyzing more precisely in several cases of the sheet material arrangement, the interlock strength was so large that the rivet was significantly rotated, and it was pulled-out on one side (photo 1 in Figure 15a–b and Figure 16a–c). In a few cases where the rivet was rotated and pulled out on one side, significant deformation of the embossment occurred (photo 3 in Figure 16c). In the remaining cases, radial cracks appeared on the embossments (photo 3 in Figure 14a–c and Figure 15a–c).

The strength of specific areas of the joint and the possibility of further deformation with the simultaneous strengthening of the sheet material significantly affect the displacement at which the maximum load capacity of the joint is achieved as well as affecting the dissipated energy. The analysis of the specific values of energy absorption by the joint during the tensile shear test will be presented in the next section of the manuscript.

Tensile shear test diagrams for individual sheet material arrangements were presented for each arrangement group. For the combination of sheet material with AW 5754-H11 and AW 6082, the highest load capacity was obtained for a homonymous arrangement of sheets (Figure 14a). The change of the material of the upper sheet from AW 5754-H11 to AW 6082 resulted in the achievement of a lower joint resistance for such joints, and a different displacement for the maximum shear force. In the case of using a sheet metal system, the upper AW 5754-H11 and the lower AW 6082 caused a further reduction of the joint load capacity, compared with the first sheet arrangement. The maximum force for the last mentioned arrangement was obtained for the smallest value of displacement (*s*), from among the group of the AW 5754-H11 and AW 6082 sheet arrangements. The difference between the maximum tensile shear force *F_s_* for the analyzed combinations of sheets was 495 N.

For the second group of AW 5754-H22 and AW 6082 material arrangements, the highest load capacity was achieved not as in the case of the identical arrangement of the sheets, but when the upper sheet was made of the AW 6082 material and the bottom of AW 5754-H22 (Figure 14b, Figure 15b and Figure 16b). For the same case of the joint, the maximum value of the tensile shear force Fs was obtained for the largest displacement s of the tested joint arrangements in this group. The lowest force of the maximum load capacity (1550 N) was obtained for the AW 5754-H22 and AW 6082 arrangement (Figure 15b). The use of AW 5754-H22 allowed to increase the load capacity of the joint, when the upper sheet was made of the AW 6082 material. The reverse arrangement of the sheets resulted in a reduction of the maximum load capacity, compared with the identical material arrangement of the sheets of aluminum alloy 5754-H22. The difference between the maximum load capacity and the minimum in this case of a different sheet arrangement was 485 N (a value similar to that obtained in the first group of material combinations).

The use of AW 5754-H24 and AW 6082 sheets resulted in large differences in the shear diagrams and in the displacement s for the maximum load capacity of the tensile shear force *F_s_* (Figure 14c, Figure 15c and Figure 16c). When analyzing individual variants, it can be seen that for the same sheets, arrangement of AW 5754-H24, the lowest displacement s was obtained, at which the maximum load capacity of the connection was achieved (Figure 16c). Moreover, the curve shows an increase in force and a slightly decrease in joint stiffness. For arrangement no.8 (AW 5754-H24 with AW 6082), the curve in the area where the maximum load capacity is reached is flatter. In this case, the maximum load capacity was obtained for a greater displacement than in the case of arrangement no.7. Completely different diagrams (Figure 16c) in joint tensile shear tests, in which the upper sheet was made of AW 6082 material, with higher mechanical properties, were obtained for arrangement no.9. For that sheet combination, a much greater displacement s was obtained, which resulted in the maximum load capacity of the joint. Among the three sheet arrangements in the third group of arrangements for sample no.9, the highest value of the joint tensile shear force was obtained.

The tensile shear test diagrams for the AW 5754 with different state conditions were presented in Figure 14a–c. The initial hardness of the rivet was 345 HB. By pressing the rivet into the joined sheets, the sheet material and the rivet were deformed. The AW 5754-H11 alloy was characterized by the lowest hardness value of 52 HB and the highest exponent of the hardening curve (n = 0.284). This means that it was highly hardened with increasing plastic deformation. Hence, for samples made of this material, the joint had the highest maximum load capacity (Figure 14a). The 5754 aluminum alloy in the H22 state condition was characterized by a 21% higher initial hardness than the AW 5754-H11. The exponent of the hardening curve was 32% lower, and the yield strength, compared with the material with the H11 state condition, was 16% higher. The material in the H24 state condition had the smallest possibilities of strengthening. This material (AW 5754-H24) had a yield strength lower, by 11%, compared with the AW 5754-H11. However, during the joining process, the AW 5754-H24 material was the fastest yielded. For this material, the lowest strengthening was obtained; hence, the maximum load capacity of the joint was obtained (Figure 14c).

In each group of the sheet material arrangement (upper/lower), the average of the maximum load capacity of the joints from five tests and the mean deviation were calculated. The results obtained in this way were presented in Table 3. The highest joint load capacity for homonymous sheet arrangements was achieved for the AW 5754 in H11 state condition (arrangement no.1). In the case of the remaining two groups of sheet material arrangement, the highest tensile shearing force of the joint was achieved when the upper sheet was made of a different material, i.e., aluminum alloy 6082 in the T6 state condition. The highest value of tensile shear force (2.05 kN) was achieved in the case of the material arrangement: the top and bottom sheet of the 5754-H11 aluminum alloy (Table 3). When the AW 5754-H22 sheet material was used, the maximum tensile shear force was lower, 1.85 kN, which was lower by 9.75%, compared with the joint for AW 5754-H11. For arrangement of both AW 5754-H24 sheets, the maximum tensile shear force was 1.35 kN (a value lower by 34%, compared with the arrangement of AW 5754-H11).

### 3.3. Interlock Parameters of CR Joints

**Figure 17:**
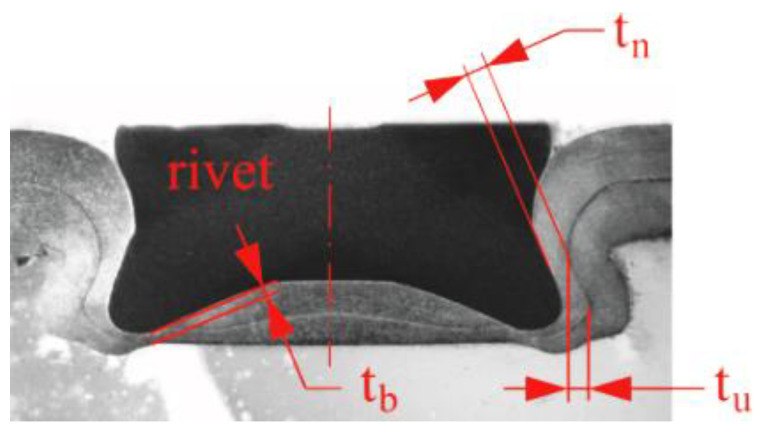
The CR joint parameters.

For SPR joints, the parameters of the interlock are defined after the rivet pierced the upper sheet [39]. An important feature of the CR joints is that the additional rivet deforms in sheets without piercing. For this type of joints, the more important interlock parameters should be defined. In the case of ClinchRivet joints, the parameters determining the strength of the joint are the geometric parameters of the interlock (*t_n_* and *t_u_*) and the minimum thickness of the material in the embossment (*t_b_*)—Figure 17. The parameters presented in Figure 17 were determined for each sheet arrangement—Table 4. 

In the group of sheet arrangements for the same material (no.1, 4 and 7 from Table 2), the highest value of the interlock (*t_u_*—size of the blocking the upper sheet in the lower sheet) was obtained for the sheet arrangement no.7. For the AW 5754-H24 sheet arrangement, the interlock parameter *t_u_* was 0.91 mm, which was greater than that obtained for the H11 state condition by 59.65%, and by 68.52%, compared with the H22 state condition. Thus, the values are comparable, and it can be concluded that the greatest influence of the sheet state condition on the size of the interlock was in the case of AW 5754-H24. Unfortunately, the larger the interlock parameter was, the smaller was the dimension *t_n_* = 0.12 mm for *t_u_* = 0.91 mm (sheet arrangement no.7). The thickness of the interlock in the upper sheet *t_n_* was smaller when the rivet was pressed deeper in the lower sheet. A high values of the interlock *t_u_* were obtained with a relatively low value of *t_n_* parameter. The greater the rivet pressing depth in the joined sheets was, the greater was the interlock, but also the smaller the values of the parameter *t_b_* were. Too big a rivet depth pressing in lower sheet caused a large pushing of the embossing material in the radial direction. Thus, there was a large thinning of the sheet material in the lower part of the embossing.

In the case of the sheet arrangement no.3 (AW 6082 joined with AW 5754-H11), the size of the interlock *t_u_* was 0.37 mm, and the *t_n_* was 0.16 mm. The change of the sheet state condition from H11 to H22 resulted in obtaining similar values of the parameters *t_u_* and *t_n_* (0.38 mm and 0.37 mm). The values of the t_b_ interlock were similar. The value of *t_n_* parameter increased by 137%. The use of the AW 5754-H24 as a lower sheet caused the *t_n_* value to increase by 106%, with the *t_u_* increasing by 46% and the *t_b_* decreasing by 15%.

**Figure 18:**
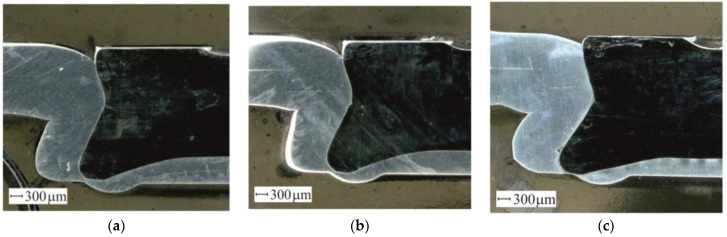
The joint cross-sections: (**a**) arrangements no.1 (AW 5754-H11 with AW 5754-H11), (**b**) arrangements no.4 (AW 5754-H22 with AW 5754-H22), (**c**) arrangements no.7 (AW 5754-H24 with AW 5754-H24).

The cross-sections were made for all sheet arrangements. An exemplary view of the CR joint was shown in Figure 18. The movable segments gradually moved outwards during the joint formation (as shown in Figure 4c). After the segment rotated, the support distance from the bottom side of the sheet changed. The deformed material was bending greater for the AW 5754-H24 sheet arrangement (Figure 18c), compared with that of AW 5754-H22 (Figure 18b). Plastic flow of the rivet material within its lower part caused the deformation of the sheet material. The smallest rivet displacement was obtained for the AW 5754-H11 sheet arrangements. Hence, the material displacement was the smallest (of the three sheet material arrangements); thus, the displacement of the movable segments did not cause a large deflection of the sheet from the die side (Figure 18a).

**Figure 19:**
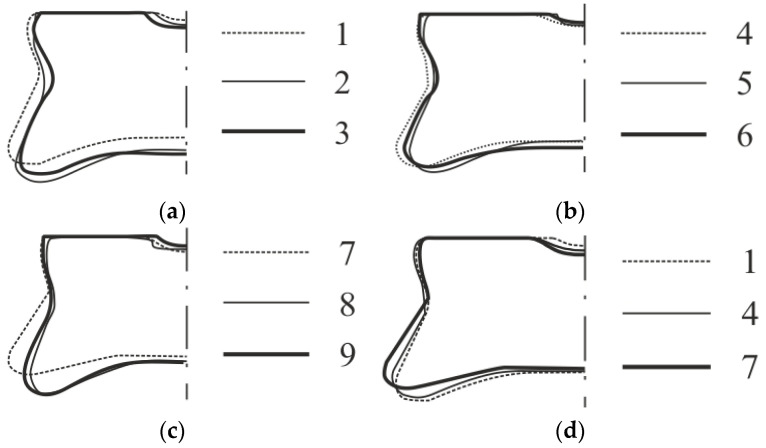
The rivets profiles for sheet arrangements in accordance with Table 2: (**a**) no.1, 2 and 3, (**b**) no.4, 5 and 6, (**c**) no.7, 8 and 9, (**d**) no.1, 4 and 7.

The specific arrangement of the sheet material during the rivet pressing process (joint forming) was responsible for the deformation of the rivets. Depending on the arrangement of the material of the upper and lower sheets, the rivet was subject to various deformations, which resulted in its final interlocking in the joined sheets (Figure 19). The greatest effect of changing the sheet material arrangement on the rivet deformation was observed in the third group of combinations (Figure 19c). The use of replacing one of the material sheets (upper or lower) of the 5754 aluminum alloy with AW 6082 significantly changed the shape of the rivet in the pressed joint. The greatest displacement of the rivet material in the radial direction (forcing the material to flow in the areas between the fixed segments) was obtained for a homonymous material system of AW 5754 sheets with three different state conditions. The use of AW 6082 for the upper or lower sheet resulted in greater sheet flow resistance, which resulted in less interlocking of the joined sheets (Table 4). The parameters of the interlock (*t_n_* and *t_u_*) and the smallest thickness at the bottom (*t_b_*) of the embossment were presented in Table 4. The use of a combination of the 6082 and 5754 aluminum alloys limited the radial flow of the rivet material, and forced the flow of sheet material under the rivet (Figure 19). Comparing the rivet contour after the joint was formed, it was observed that the biggest deformation of the rivet and the radial flow of the rivet material were obtained for a homonymous arrangement of the sheet material (Figure 19). The AW 5754-H24, with a hardness of 70 HB but with the lowest hardening index n = 0.014, caused the lowest flow resistance and the greatest displacement perpendicular to the rivet axis (Figure 19—arrangement no.7).

### 3.4. Dissipated Energy

**Figure 20:**
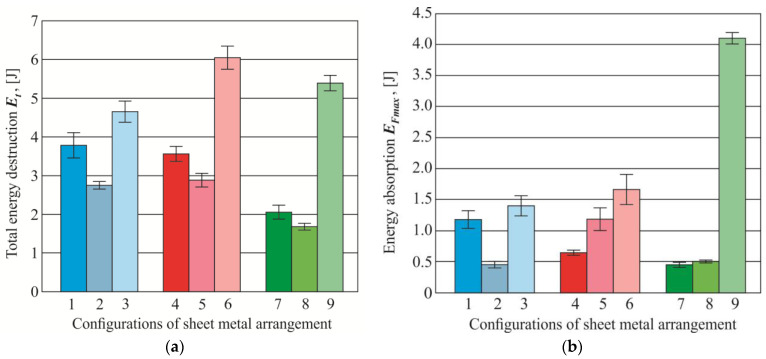
The dissipated energy: (**a**) up to fracture, (**b**) up to the maximum tensile shear force.

The dissipated energy up to fracture calculated from joint tensile shear test diagrams was presented in Figure 20a. The total energy of failure does not result from the maximum destructive force (Figure 14, Figure 15 and Figure 16). In the case of joint loading, the linear part is important (in terms of linear deformation—up to the yield point). In the case of thin-walled structures, the elastic part is important, and in the case of motor vehicle parts, the amount of energy absorption is an important parameter. However, the ISO 12996: 2013 standard [36] defines the amount of dissipated energy up to fracture determined on the tensile shear test (Equation (2)). According to this ISO standard, for this type of joint (CR joints), the dissipated energy up to maximum tensile shear force can be calculated (Equation (1)). It allows to indirectly determine the shear force-displacement diagram until the maximum load capacity of the joint is achieved under the influence of the tensile shear load)—Figure 20b. Among the investigated sheet arrangements, the highest values in the arrangement groups were obtained when the upper sheet was made of 6082 aluminum alloy and the bottom one was AW 5754. On the other hand, the highest dissipated energy up to maximum tensile shear force was obtained for the arrangement no.9 (AW 6082 and AW 5754-H24, according to Table 2).

## 4. Conclusions

Based on experimental joining of aluminum alloy sheets (AW 5754 in different state conditions and AW 6082-T6) by using ClinchRivet joining technology and on tensile shear tests of CR joints, the following conclusions can be formulated:

- Sheet material arrangements influence the material continuity of the embossment—for upper sheet AW 6082-T6, no cracks in the interlock area were obtained.
- Sheet material arrangements influence the forming force—the highest differences in forming force values were obtained for joining AW 6082-T6 with AW 5754 in different state conditions (H11, H22 and H24).
- Sheet material arrangements influence the tensile shearing force of the joint—the highest value was achieved when the upper sheet was made AW 6082-T6.
- The interlock parameter *t_u_* for AW 5754 in H24 state conditions was bigger by 59.65%, compared with H11, and to H22 by 68.52%.
- The deformation of the additional rivet strongly influences the interlock parameters t_b_.
- The biggest deformation of the rivet and the radial flow of the rivet material were obtained for a homonymous arrangement of the sheet materials.

## Figures and Tables

**Table 1:** Mechanical properties and state conditions of the aluminum alloy sheets.

Material	State Condition	Yield Strength Re [MPa]	Tensile Strength Rm [MPa]	Elongation after Fracture A80 [%]	Brinell Hardness [HB]	Strain Hardening Exponent n	Sheet Thickness [mm]
AW 5754	H11	143	232	19.7	52	0.284	0.8
	H22	166	251	13.9	63	0.193	
	H24	127	231	4.8	70	0.014	
AW 6082	T6	314	342	13.5	90	0.087	0.9

**Table 2:** Sheet arrangements.

Arrangement Denotation	Sheet Material	
	Top Sheet	Bottom Sheet
1	AW 5754-H11	AW 5754-H11
2	AW 5754-H11	6082-T6
3	6082-T6	AW 5754-H11
4	AW 5754-H22	AW 5754-H22
5	AW 5754-H22	6082-T6
6	6082-T6	AW 5754-H22
7	AW 5754-H24	AW 5754-H24
8	AW 5754-H24	6082-T6
9	6082-T6	AW 5754-H24

**Table 3:** Parameters of joint strength from tensile shear test.

Joint Strength Parameters		Joint Sheets Arrangements								
		1	2	3	4	5	6	7	8	9
Maximum shear force	F_smax_ [N]	2011	1528	1864	1846	1576	2016	1336	1064	1465
Standard deviation of F_smax_	s [N]	100	70.4	97.4	29.2	26.2	60.1	37.6	19.5	21.3
Coefficient of variation of F_smax_	C_v_ [%]	5.0	4.6	5.2	1.6	1.7	3.0	2.8	1.8	1.5
Displacement at the maximum load	S_Fmax_ [mm]	0.52	0.41	0.75	0.47	0.87	1.15	0.38	0.57	3.55
Displacement up to fracture	S_Fracture_ [mm]	2.48	2.45	2.82	2.52	2.32	3.76	2.16	2.26	4.64

**Table 4:** CR joint interlock parameters.

Sheets Arrangement									
Parameter [mm]	1	2	3	4	5	6	7	8	9
*t_n_* (top thickness)	0.18	0.44	0.16	0.23	0.23	0.38	0.12	0.05	0.33
*t_u_* (sheets interlock)	0.57	0.65	0.37	0.54	0.27	0.37	0.91	0.50	0.54
*t_b_* (bottom thickness)	0.10	0.27	0.26	0.12	0.25	0.26	0.05	0.15	0.22

## Data Availability

Research data can be obtained from the author.
